# Chronic Exercise as a Modulator of Cognitive Control: Investigating the Electrophysiological Indices of Performance Monitoring

**DOI:** 10.3389/fpsyg.2022.814199

**Published:** 2022-04-05

**Authors:** Meaghan L. Wunder, W. Richard Staines

**Affiliations:** Department of Kinesiology and Health Sciences, University of Waterloo, Waterloo, ON, Canada

**Keywords:** attention, chronic exercise, event-related potentials, performance monitoring, action monitoring

## Abstract

Exercise may influence components of executive functioning, specifically cognitive control and action monitoring. We aimed to determine whether high level exercise improves the efficacy of cognitive control in response to differing levels of conflict. Fitter individuals were expected to demonstrate enhanced action monitoring and optimal levels of cognitive control in response to changing task demands. Participants were divided into the highly active (HA) or low-active group based on self-reported activity using the International Physical Activity Questionnaire. A modified flanker task was then performed, in which the level of conflict was modulated by distance of distractors from the target (close, far) and congruency of arrows (incongruent, congruent). Electroencephalography (EEG) was collected during 800 trials; trials were 80% congruent, 20% incongruent, 50% close, and 50% far. The error-related negativity (ERN) and error positivity (Pe) were extracted from the difference wave of correct and incorrect response locked epochs, the N2 from the difference wave of congruent and incongruent stimulus locked epochs and the P3 from stimulus locked epochs. The HA group showed a larger Pe amplitude compared to the low-active group. Close trials elicited a larger N2 amplitude than far trials in the HA group, but not the low-active group, the HA group also made fewer errors on far trials than on close trials. Finally, the P3 was smaller in the lowest conflict condition in the HA, but not the low-active group. These findings suggest that habitual, high levels of exercise may influence the endogenous processing involved in pre-response conflict detection and the post-error response.

## Introduction

An important aspect of human cognitive control is the ability to monitor action and minimize undesirable responses. The medial frontal cortex; specifically, the anterior cingulate cortex (ACC) is thought to play a role in these processes (Botvinick et al., 2001; Gehring and Fencsik, 2001; van Veen et al., 2001; Yeung et al., 2004). The ACC is activated when detecting response conflict, especially post-error. Importantly, the level of activation can be influenced by the exertion of top-down cognitive control (Carter et al., 2000; Gehring and Knight, 2000; van Veen and Carter, 2002). Many studies use a version of a flanker task; in which participants must respond to a central target (indicating left or right), while also being presented with either incongruent (opposite) or congruent distractor stimuli (Eriksen and Eriksen, 1974) to investigate this phenomenon. van Veen and Carter (2002) found that ACC activation was maximal on incongruent trials when preceded by a congruent rather than an incongruent trial. In the latter condition, the initial high conflict trial activates control mechanisms to minimize the conflict detected by the ACC in the subsequent trial. Likewise, Carter et al. (2000) found that if 80% of trials are congruent, then less top-down control is recruited and the ACC is activated to a greater degree on high conflict or error trials. Conversely, if 80% of trials are incongruent, more top-down control is initially recruited, resulting in less ACC activation on high conflict or error trials. The top-down modulation of the ACC may be due to prefrontal associations (Gehring and Knight, 2000; Walsh et al., 2011). Specifically, the dorsolateral prefrontal cortex (dlPFC; Kerns, 2004) and the frontoparietal attentional control system (Walsh et al., 2011) are engaged after high conflict trials resulting in high ACC activation, denoting the need for increased cognitive control. Notably, the control exerted by the dlPFC on the ACC is analogous to a model depicting the salience network proposed by Menon and Uddin (2010) in which the ACC is a central node. Importantly, the salience network is responsible for dynamic switching between the default mode network and the attentional networks such as the central executive network, or frontoparietal attentional network upon the detection of emotional, homeostatic, or cognitive salience (Crottaz-Herbette and Menon, 2006; Menon and Uddin, 2010; Chen et al., 2013; Di and Biswal, 2014; Zhou et al., 2017). This network therefore provides a framework through which both bottom-up (from primary sensory areas and the insular cortex) and top-down (from prefrontal areas) control may be integrated in the ACC in response to the detection of salience, or conflict.

Furthermore, this model can be translated to an electrophysiological framework consisting of the N2 and P3 stimulus locked event-related potentials (ERPs) and the error-related negativity (ERN) and error positivity (Pe) response locked components on error trials. These electrophysiological markers have been used extensively to investigate performance and error monitoring. ERPs extracted from electroencephalography (EEG) provide information regarding endogenous processes occurring during stimulus identification and response-selection. The N2, P3, ERN, and Pe ERP peaks are specifically relevant to the recruitment of cognitive control and error processing.

The ERN is an ERP which appears selectively on error trials and has been localized to the dorsal ACC (Dehaene et al., 1994; Krigolson et al., 2008). It is an indicator of ACC activation and conflict monitoring after an error; specifically, *outcome errors* or *slips*; motor errors representing a failure to achieve the desired response (Dehaene et al., 1994; Krigolson et al., 2008). The ERN is characterized by a sharp negative deflection which is largest over frontocentral electrodes. It appears shortly after EMG onset of the limb performing an error and will peak approximately 100 ms later (Gehring et al., 1993). Various experimental manipulations have been found to alter the amplitude of the ERN. Notably, amplitude tends to increase when emphasis is placed on accuracy (Gehring et al., 1995) or when the error made is costlier (Gehring et al., 1993). The Pe is another error-related component, thought to reflect conscious awareness of the commission of an error. It has been dissociated from the ERN and localized to the rostral ACC (van Veen and Carter, 2002). The Pe and the ERN are therefore thought to reflect different systems of error monitoring. The Pe has been associated with later accumulation of evidence suggesting that an error has been made, perhaps occurring in working memory. This processing is thought to receive input from cognitive, autonomic, and sensory areas to evaluate the certainty of error commission (Gregorio et al., 2018).

Furthermore, the N2 is a stimulus locked fronto-central negative deflection occurring approximately 200–350 ms after stimulus delivery. The anterior N2 subcomponent specifically, is associated with pre-response conflict and is generally larger in amplitude on more incongruent trials (Yeung et al., 2004; Folstein et al., 2008). The N2 has been dissociated from the ERN (Iannaccone et al., 2015) and is a marker of the need for cognitive control during certain cognitive tasks (Folstein et al., 2008). Finally, the P3, like the N2, is a stimulus locked ERP component occurring approximately 300–800 ms after stimulus delivery. It is thought to reflect strategic “context updating” rather than tactical processing, as it frequently occurs too late to impact a response. In this sense, “context” refers to the overall state of the environment (Donchin, 1981). Consistent with this explanation, the P3 is generally larger when stimulus discrimination requires more effort, or when response inhibition is required; perhaps behaving as a marker of attentional resource allocation (Isreal et al., 1980; Groom and Cragg, 2015). The N2 and P3 will therefore characterize the endogenous response to conflict and recruitment of cognitive control.

Evidence suggests that aerobic exercise may modulate these neuroelectric indices. Themanson et al. (2008) investigated the ability to flexibly implement appropriate control in response to task demands using a flanker paradigm when stressing accuracy or speed. Using the ERN and post-error accuracy as indexes of action monitoring, they found that more fit individuals displayed larger ERN amplitudes and greater post-error accuracy on conditions stressing accuracy, but not on conditions stressing speed. High fitness was also related to greater differences in ERN amplitude across task conditions, leading researchers to suggest that cardiorespiratory fitness may be related to increased flexibility in the modulation of cognitive control regarding task demands. Likewise, when stressing speed, multiple studies have shown a smaller amplitude ERN in more aerobically fit participants (Themanson et al., 2006; Themanson and Hillman, 2006; Hillman et al., 2008, 2009). Larger amplitude markers of cognitive operations regarding the allocation of attentional resources such as the P3 (Hillman et al., 2008; Kao et al., 2020) and the Pe (Hillman et al., 2009) have also been observed in more aerobically fit participants. Furthermore, the above studies have not observed differences attributed to fitness regarding N2 amplitude. It is important to note that these studies did not modify their behavioral tasks to employ alternative sources of conflict, the sole conflict modulation was therefore the congruency of the target relative to distractors during flanker paradigms. Interestingly, when trial to trial adaptations were analyzed by Kamijo and Takeda (2013), they found that more fit participants demonstrated a larger amplitude N2 on incongruent trials that were preceded by congruent trials. Likewise, Danielmeier et al. (2009) modulated the distance between the distractors and the target to modulate the level of conflict. They did not account for fitness level, but nonetheless found larger amplitude N2 components when the distractors were closer to the target, reflecting increased conflict. Additional conflict modulation beyond the classic flanker paradigm is therefore probably necessary to observe any N2 modulation attributed to fitness.

Additionally, the above reports have suggested numerous mechanisms by which physical activity may be modifying these electrophysiological signals. Notably, aerobic exercise seems to prime the brain to create an ideal environment for use dependent learning and plasticity (Hötting and Röder, 2013; Raichlen et al., 2016; Raichlen and Alexander, 2017). Habitual activation of neural systems combined with the cognitive demands of exercise may lead to long term cortical changes, reinforcement of these pathways may in turn manifest as modulated electrophysiological signals during the cognitive tasks previously described. The present study therefore sought to investigate the effects of habitual high levels of exercise [as defined by the International Physical Activity Questionnaire (IPAQ, 2002)] on neuroelectric indices of action monitoring during a modified flanker task where, like Danielmeier et al. (2009), conflict was modulated by distance as well as congruency. This modulation was in place to allow for a more robust analysis of the effect of conflict. For example, as the N2 is extracted from the difference wave of congruent and incongruent trials the additional distance modification allows for subsequent analysis of the effect of conflict. It was hypothesized that the highly active (HA) group would demonstrate shorter response times and make fewer errors, additionally, error commission would be modulated by conflict level to a greater degree than their low-active counterparts. Given the proportionality of congruent vs. incongruent trials (80% congruent, 20% incongruent), it was hypothesized that the HA group would show smaller amplitude N2 ERP components across all trials as compared to the low-active group. Upon the commission of an error, the HA group was hypothesized to demonstrate a smaller amplitude ERN than the low-active group, and a subsequently larger amplitude Pe. This reflects minimal activation due to response conflict and subsequently enhanced recruitment of attentional resources when needed. In summary, more fit individuals were expected to demonstrate enhanced action monitoring in response to high conflict (incongruent) trials, and less activation of attentional networks due to the high frequency of low conflict (congruent) trials; they were expected to adapt to these conditions more efficiently than their low-active counterparts.

## Materials and Methods

### Participants

Twenty participants were recruited into one of two groups; 10 in the HA group (mean age ± SE: 20.9 ± 0.41, three male) and 10 in the low-active group (mean age: 20.7 ± 0.47; one male). Criteria for inclusion in the HA group required a high classification on the International Physical Activity Questionnaire (IPAQ; 2002). This questionnaire assesses the time spent walking, doing moderate intensity activity and doing vigorous intensity activity across four domains (work, transportation, house-work, and leisure). This information is then used to calculate the approximate number of metabolic equivalents (METS) the individual expends per week. A high classification requires either (a) vigorous intensity activity on at least 3 days and accumulating at least 1,500 MET-minutes per week or (b) 7 or more days of any combination of walking, moderate and vigorous intensity activities accumulating at least 3,000 MET-minutes per week. Inclusion in the low-active group requires that individuals be in the low classification on the IPAQ (2002). Either no activity is reported, or the activity reported is not enough to meet the criteria for moderate or high classification. Specifically, participants in the “low” classification expended less than 600 MET-minutes per week. Validity of this questionnaire has been reported to be similar to other self-reported physical activity questionnaires and the stability of the reliability has been deemed acceptable in college students (Dinger et al., 2006) and in individuals aged 18–65 across 12 countries including Canada (Craig et al., 2003). Participants in the study must also have self-reported a consistent level of activity or inactivity over the previous 8 months. Participants were excluded if they had any underlying neurological illness or were on any medications affecting the central nervous system. The experimental procedures were approved by the Office of Research Ethics at the University of Waterloo and carried out in accordance with the Declaration of Helsinki.

### Behavioral Task

**Figure 1:**
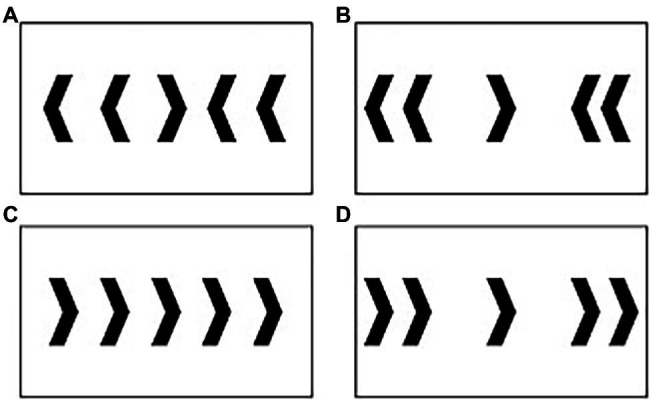
Trials included in the modified Flanker task: **(A)** close-incongruent; **(B)** far-incongruent; **(C)** close-congruent; and **(D)** far-congruent. Note that this figure only shows trials indicating a right click, though right vs. left arrows were displayed in equal proportion during the task.

A speeded modified computer-based flanker task was employed using Stim 2 (Compumedics Neuroscan, Charlotte, NC, United States). This task required participants to respond as quickly and accurately as possible to the central target arrow. The target arrow pointed either to the left or to the right, denoting a left or right mouse click with the right hand as a response. Four flanking distractor arrows were presented on either side of the target. These arrows differed in respect to congruency and distance relative to the target; close and far arrows flanking the target arrow were 2.5 and 8.5 cm away from the target, respectively. Figure 1 illustrates the four trial types: (A) close-incongruent; (B) far-incongruent; (C) close-congruent; and (D) far-congruent. Importantly, close-incongruent trials reflect maximal interference and conflict, while far-congruent trials reflect minimal interference and conflict.

The task was presented over four blocks of 200 trials each (800 trials total). The task consisted of approximately 80% congruent and 20% incongruent trials; 50% close and 50% far (310 close-congruent, 310 far-congruent, 90 close-incongruent, and 90 far-incongruent trials total). A proportionality of 80% congruent has been shown to engage less strategic processing and elicit even more conflict and top-down control on incongruent trials (Carter et al., 2000). This method provides a clear relationship between relative stimulus interference, control requirements and conflict detection. The stimuli were displayed for 200 ms followed by a blank white screen with an inter-trial interval randomized between 1,200 and 1,400 ms.

### Electrophysiological Monitoring

Electroencephalography data were recorded from 15 electrode sites (FP1, FZ, FCZ, CZ, CPZ, PZ, OZ, F3, F4, FC3, FC4, C3, C4, P3, and P4; 32 channel Quik-Cap, Neuroscan, Compumedics, NC, United States) in accordance with the International 10–20 System for electrode placement and referenced to the linked mastoids. Impedance was maintained less than 5 kΩ. Data were collected with a DC–100 Hz filter and digitized at a sample rate of 500 Hz (SynAmps 2, Neuroscan 4.5, Compumedics, United States). Continuous data were then epoched to be either stimulus- or response locked and baseline-corrected to the pre-stimulus/response interval. Stimulus locked data were used to extract the N2 and P3 ERP components and epochs were taken from 100 ms pre-stimulus to 600 ms post-stimulus (Groom and Cragg, 2015). The N2 was extracted by subtracting congruent trial epochs from incongruent trial epochs and detecting the negative peak occurring between 200 and 350 ms. The N2 for far trials was measured separately from close trials to quantify varying levels of conflict between the conditions. The N2 was extracted from frontocentral electrodes and was maximal at FCZ, consistent with previous studies (Groom and Cragg, 2015). The P3 was extracted by detecting the positive deflection peaking between 300 and 500 ms on stimulus locked epochs. The P3 was measured for close-congruent, close-incongruent, far-congruent, and far-incongruent trials separately in order to provide an indication of attentional resource allocation for each condition. The P3 was extracted from central electrode sites (FZ, FCZ, CZ, CPZ, and PZ) and was maximal at CZ. Response locked epochs were taken from 100 ms pre-response to 600 ms post-response. The ERN was extracted from response locked epochs by subtracting correct from incorrect trial epochs and detecting the negative peak occurring between 0 and 150 ms post-response. Close vs. far trials were subsequently measured separately. Finally, the Pe was extracted from the same difference wave as the ERN and was defined as the maximum peak occurring between 100 and 300 ms, close vs. far trials were likewise measured separately. Both the ERN and Pe were extracted from electrode FCZ as they were both maximal at this location consistent with previous studies (Gehring and Fencsik, 2001; Krigolson et al., 2008).

### Data Analysis

Mean physical activity level difference between groups (HA and low-active) was assessed using a one-tailed *t*-test comparing average MET-minutes expended per week. Next, 2 × 2 mixed model ANOVAs with the factors GROUP (HA, low-active) and CONFLICT (close, far) were used for the analysis of the ERN, Pe and N2 ERP components as well as behavioral data (errors made and response time). Further, planned contrasts were run to test the specific hypothesis related to error modulation due to conflict level in both groups; that error commission would be modulated by conflict level to a greater degree in the HA group. Finally, a 2 × 4 mixed model ANOVA with the factors GROUP (HA, low-active) and CONFLICT (close-congruent, close-incongruent, far-congruent, and far-incongruent) was used for the analysis of the P3 ERP component. *Post-hoc* Tukey analyses were used to elucidate any additional significant differences identified from the ANOVAs. Data sets were assessed for normality and homogeneity of variance to ensure that the assumptions for performing the ANOVA were upheld. Statistical analysis was conducted with SAS and significance was taken as *p* < 0.05.

## Results

### Participants and Behavior

Participants’ MET minutes expended per week were significantly different between the HA group (*M* = 4991.75 MET min/week, SE = 637.13) and the low-active group (*M* = 452.65 MET min/week, SE = 34.47; *t*_18_ = 7.12, *p* < 0.001); demonstrating a distinct difference in activity level between the two groups.

**Figure 2:**
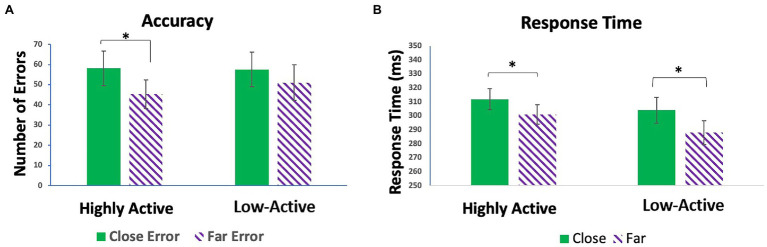
**(A)** Mean number of errors made on close vs. far trials in the highly active (HA) and low-active groups. **(B)** Mean response times on close vs. far trials in the HA and low-active groups. Error bars reflect SE; *denotes *p* < 0.05.

There was no significant effect of GROUP (*F*_1,18_ = 0.98, *p* = 0.335) on response time; however, there was a significant effect of CONFLICT (*F*_1,18_ = 62.68, *p* < 0.001). Participants took more time to respond to close than far trials. There was no GROUP*CONFLICT interaction (*F*_1,18_ = 2.98, *p* = 0.101) on response time. Likewise, there was no significant effect of GROUP (*F*_1,18_ = 0.05, *p* = 0.824) on number of errors made. There was, however, a significant effect of CONFLICT (*F*_1,18_ = 18.09, *p* < 0.001). There was no GROUP*CONFLICT interaction (*F*_1,18_ = 1.85, *p* = 0.191). Contrasts demonstrated that significantly more errors were made on high conflict (close) trials than low conflict (far) trials in the HA group (*t*_1,18_ = 3.97, *p* < 0.001). This relationship was not as robust, and non-significant in the low-active group (*t*_1,18_ = 2.05, *p* = 0.056), showing that error commission was modulated by conflict to a greater degree in the HA group than the low-active group (Figure 2).

### Event-Related Potentials

#### Error-Related Negativity

**Figure 3:**
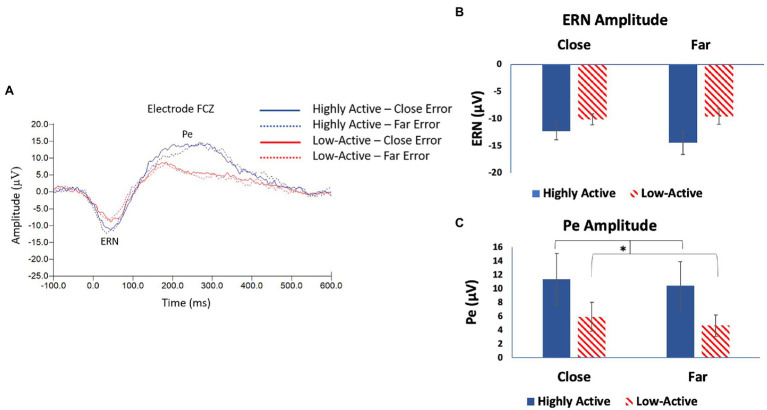
**(A)** Electroencephalography (EEG) grand average response locked waveforms taken from electrode FCZ; the difference wave between correct and error trials showing the error-related negativity (ERN) and error positivity (Pe) event-related potential (ERP) components. **(B)** Mean ERN amplitude taken from difference wave—FCZ. **(C)** Mean Pe amplitude taken from difference wave—FCZ. Error bars reflect SE; *denotes *p* < 0.05.

The ERN was measured from electrode FCZ (Figure 3A) as it was maximal across participants at this location (mean ± SE number of epochs included per participant: HA group, close condition = 56 ± 8; HA group, far condition = 44 ± 7; low-active group, close condition = 58 ± 9; low-active group, far condition = 51 ± 9; and Mean latency: 44.9 ± 3 ms). As demonstrated in Figure 3B, there was no significant main effect for GROUP (*F*_1,17_ = 2.8, *p* = 0.11) or CONFLICT (*F*_1,16_ = 3.1, *p* = 0.1) on ERN amplitude, nor was there any significant interaction of GROUP*CONFLICT (*F*_1,16_ = 1.7, *p* = 0.2). This was not in accordance with our hypothesis that the HA group would demonstrate a smaller amplitude ERN than the low-active group across trial type, as there was no statistical difference.

#### Error Positivity

The Pe was measured from the same ERP trace taken from FCZ as the ERN (Figure 3A), as it was maximal across participants at this location (mean number of epochs included were the same as those included in the ERN; Mean latency: 194.1 ± 8.2 ms). There was a significant main effect of GROUP on Pe amplitude (*F*_1,16_ = 6.6, *p* = 0.02). As seen in Figure 3C, the Pe was larger in amplitude after close error trials in the HA group than in the low-active group; and after far-error trials in the HA group than the low-active group. There was no significant effect of CONFLICT (*F*_1,15_ = 0.8, *p* = 0.4) or interaction of GROUP*CONFLICT (*F*_1,15_ = 0.7, *p* = 0.4). These results are in accordance with our hypothesis that the HA group would demonstrate larger amplitude Pe components than the low-active group after the commission of an error.

#### N2

**Figure 4:**
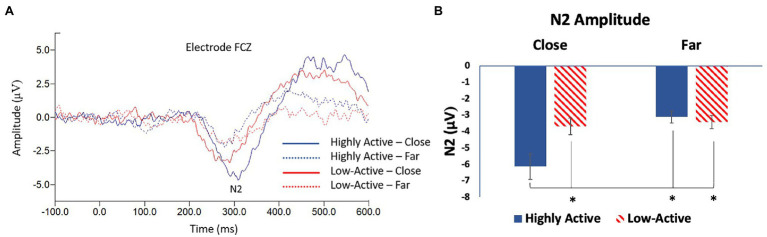
**(A)** Grand average stimulus locked waveform taken from electrode FCZ; the difference wave between incongruent and congruent trials showing the N2 ERP component. **(B)** Mean N2 amplitude—FCZ. Error bars reflect SE; *denotes *p* < 0.05.

The N2 was measured from the FCZ electrode (Figure 4A) as it was maximal across participants in this location (mean ± SE number of epochs per participant: HA group, close condition = 89 ± 1; HA group, far condition = 82 ± 1; low-active group, close condition = 87 ± 2; low-active group, far condition = 82 ± 2; Mean latency: 298.8 ms). As illustrated in Figure 4B, there was a significant main effect of CONFLICT on N2 amplitude (*F*_1,16_ = 14.2, *p* = 0.002), and a significant GROUP*CONFLICT interaction (*F*_1,16_ = 9.7, *p* = 0.007). There was no significant effect of GROUP (*F*_1,18_ = 2.8, *p* = 0.1). *Post-hoc* Tukey tests revealed that the HA group showed a larger amplitude N2 during high conflict (close) trials than during low conflict (far) trials; the HA group’s N2 during close trials was also significantly larger than the low-active group during high conflict (close) trials or low conflict (far) trials. These results were not strictly in line with our hypothesis that the HA group would demonstrate a smaller amplitude N2 across trials, though they do reflect differential modulation of the N2 in the HA group due to conflict that was not demonstrated in the low-active group.

#### P3

**Figure 5:**
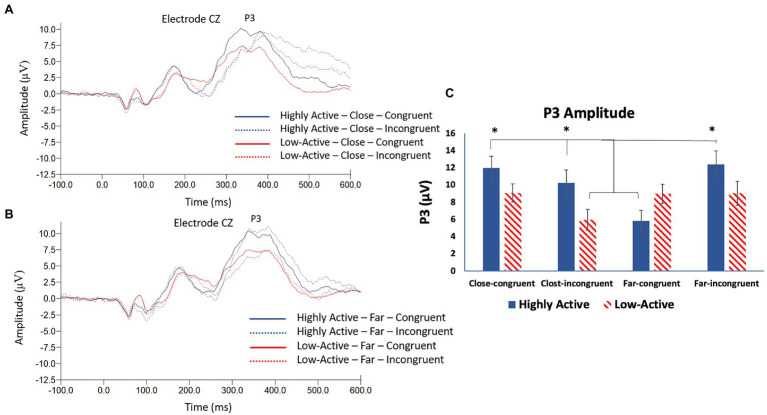
**(A)** Grand average stimulus locked waveform taken from electrode CZ depicting the P3 ERP component in both groups during close, congruent, and incongruent trials. **(B)** Grand average stimulus locked waveform taken from electrode CZ depicting the P3 ERP component in both groups during far, congruent and incongruent trials. **(C)** Mean P3 amplitude—CZ. Error bars reflect SE; *denotes *p* < 0.05.

The P3 was measured from the CZ electrode (Figures 5A,B) as it was maximal across participants at this location (mean ± SE number of epochs per participant: HA group, CC condition = 297 ± 3; HA group, CI condition = 89 ± 1; HA group, FC condition = 305 ± 2; HA group, FI condition = 82 ± 1; low-active group, CC condition = 296 ± 3; low-active group, CI condition = 87 ± 2; low-active group, FC condition = 308 ± 2; low-active group, FI condition = 82 ± 2; and Mean latency: 298.8 ms). As illustrated in Figure 5C, there was a significant main effect of CONFLICT on P3 amplitude (*F*_3,17_ = 7.4, *p* < 0.001), and a significant GROUP*CONFLICT interaction (*F*_3,17_ = 7.5, *p* < 0.001). There was no significant effect of GROUP (*F*_1,17_ = 1.4, *p* = 0.3). *Post-hoc* Tukey tests revealed a larger amplitude P3 component in the HA group during close-congruent, close-incongruent, and far-incongruent trials than the HA group during far-congruent trials and the low-active group during close-incongruent trials. There were no significant differences regarding the low-active group during close-congruent, far-congruent, or far-incongruent trials. In other words, the HA group showed a smaller P3 amplitude on trials reflecting minimal conflict (far-congruent); this trend was not replicated in the low-active group.

## Discussion

The present study aimed to elucidate differences in neural markers of performance and conflict monitoring between individuals who are highly active and those who are not. There was no significant difference between groups regarding response time or total errors made, though only the HA group made significantly fewer errors on low (far) compared to high (close) conflict trials. Furthermore, there was no group difference regarding ERN amplitude; however, the HA group did display different patterns of neuroelectric modulation in response to conflict level not demonstrated by the low-active group. The HA group displayed a larger amplitude Pe on error trials; this pattern of Pe amplitude modulation was not replicated in the low-active group. The HA group also demonstrated a larger amplitude N2 on close (high conflict) vs. far (low conflict) trials, whereas the low-active group did not; importantly, the N2 elicited by close trials in the HA group was also significantly larger than the N2 elicited in either condition in the low-active group. Finally, the HA group showed a significantly smaller P3 amplitude in the far-congruent (least conflict) trials relative to all other trial types. This relationship was not replicated in the low-active group, which showed no significant difference in P3 amplitude regardless of trial type. Interestingly, the P3 elicited by close-incongruent trials (most conflict) in the low-active group was significantly smaller than the P3 elicited by the HA group during close-congruent, close-incongruent, and far-incongruent trials. Collectively, these results suggest some differences concerning the endogenous processing of conflict and errors between groups. Specifically, those partaking in HA seem to process the level of conflict or interference in an array differently than those who do not, this may allow them to better adapt to changing task demands and respond appropriately (i.e., more successfully ignoring distractor stimuli on “easier” far trials compared to close). These results also suggest that the HA group may process errors differently than their low-active counterparts. Collectively, the results of this study suggest that HA may confer some cognitive impact in terms of conflict and performance monitoring both before and after a response has been made.

### Behavioral Effects of HA

In the present investigation, no group differences in response time or accuracy were observed, contrary to our initial hypothesis. However, when errors made are divided based on conflict level, the HA group made significantly fewer errors on low conflict rather than high conflict trials. This effect did not reach significance in the low-active group. Though several studies report more correct responses and increased accuracy with greater fitness (Hillman et al., 2009; Martin et al., 2016), our findings are largely in line with results from inhibitory control tasks testing more fit vs. less fit participants. Studies have either reported no group difference in accuracy and response time (Themanson and Hillman, 2006; Themanson et al., 2008), or subtle group differences which emerge with further analysis (Themanson et al., 2006, 2008; Kamijo and Takeda, 2013). For example, Themanson et al. (2006) observed a lower switch cost related to congruency in reaction time in older, more fit participants relative to older, less fit participants. This effect was not demonstrated in their younger counterparts. Furthermore, Kamijo and Takeda (2013) showed that a more active group responded equally as quickly to incongruent trials preceded by congruent trials as incongruent trials preceded by incongruent trials, while their less active counterparts responded slower to incongruent trials preceded by congruent trials. Finally, Themanson et al. (2008) observed that when stressing accuracy during a flanker task, the higher fit group was more accurate after making an error than the less fit group. These findings suggest that any behavioral effect conferred by fitness may either be nuanced (Themanson et al., 2008; Kamijo and Takeda, 2013), emerge with age (Themanson et al., 2006), or require a more difficult task. Indeed, Cona et al. (2015) found that faster ultramarathon runners performed more accurately and quickly on trials requiring inhibitory control during a dual task paradigm than slower (less fit) runners. Nevertheless, in the present study, we showed that while the HA and low-active groups made approximately the same amount of errors, the HA group made significantly less on low conflict trials. If relative ease of target discrimination and management of interference can be inferred by number of errors made; the HA group was able to inhibit the distractors in the low conflict trials more accurately than the distractors on high conflict trials. Conversely, the low-active group was not able to adapt to the changing level of conflict and thus, inhibited the interference from all trials to the same degree; resulting in similar performance on both high and low conflict trials. These findings suggest that the HA group was better able to adapt to the changing level of conflict presented to them and respond accordingly, which is consistent with the spirit of our initial hypothesis.

### Error Monitoring

In the present study, there was no significant difference between groups or conditions in terms of ERN amplitude; however, the Pe was elevated in the HA vs. the low-active group across conditions. The ERN findings are not in accordance with our hypothesis, as we expected a smaller ERN in the HA group as compared to the low-active group. Though multiple studies have shown a manipulation of the ERN in active vs. low-active groups (Themanson et al., 2006, 2008; Themanson and Hillman, 2006; Hillman et al., 2008, 2009), it is not entirely surprising that there was no significant difference across group or condition in the present study. The ERN reflects the response conflict between the correct response representation and the actual (error) response made, a larger amplitude ERN denoting more conflict between these two representations. In the current study, regardless of the condition, the correct response was always one of two options (a left or right mouse click in response to a left or right pointing arrow). Therefore, the error made was always choosing left over right or vice versa. In this case, the ERN would therefore be elicited by the difference between the left and right arrow representations. It is therefore unsurprising that different conditions (close vs. far) did not result in differences in ERN amplitude. Furthermore, the finding that Pe was greater in the HA group than the low-active group across conditions, is consistent with our hypothesis. The Pe is a measure of the conscious awareness of the commission of an error and the accumulation and maintenance of evidence for the error in working memory (Gregorio et al., 2018). Therefore, a larger amplitude Pe denotes enhanced awareness of the error made; reflecting enhanced performance monitoring as described by this marker. The finding that the HA group showed a larger amplitude Pe than the low-active group regardless of condition therefore provides evidence that the HA group can more effectively process the errors that they make, described as superior performance monitoring, or enhanced awareness of error vs. correct trials. Furthermore, the Pe has been attributed to enhanced attentional resources toward the error, thus perhaps enhancing top-down attentional control on subsequent trials (Themanson et al., 2006). This phenomenon is unclear in the current investigation, as we did not analyze trial to trial ERP changes. Future work investigating ERP modulation across consecutive trials post-error would help to provide greater insight into this potential phenomenon.

### Performance Monitoring

Both the N2 and P3 stimulus locked ERP components showed significant differences depending on both the group and conflict level. The N2 specifically was largest in the HA group in the close (high conflict) condition relative to the far condition and the low-active group in both conditions. This finding was contrary to our hypothesis that the N2 would be of smaller amplitude across all trials but is consistent with enhanced conflict processing within the HA group. This finding is interesting as numerous studies have failed to observe modulations of the N2 in active vs. inactive participants (Themanson et al., 2006, 2008; Hillman et al., 2009). The N2 is a marker of conflict detection and pre-response inhibition (Folstein et al., 2008) and is associated with cognitive control (Iannaccone et al., 2015). The current study therefore sought to elicit modulations of the N2 by manipulating the level of conflict in a flanker task by both congruency and distance. This method was previously employed by Danielmeier et al. (2009) who observed changes in N2 amplitude when the level of interference was modulated by a distance manipulation in a flanker task. Likewise, Kamijo and Takeda (2013) observed differences in N2 amplitude when investigating trial by trial adjustments; the N2 was largest when a congruent trial preceded an incongruent trial in a flanker task. It is evident that the N2 is sensitive to the interference present during a flanker task; an important finding in the current study is therefore that the N2 was significantly larger in the close condition in the HA group. This suggests that the HA group was able to more effectively process the level of interference/conflict present during each trial and adapt accordingly. This phenomenon is bolstered by the finding that the P3 ERP component was significantly larger in high vs. low conflict trials in the HA, but not the low-active group. The P3 is an indication of strategic processing (Verleger, 1988) thought to reflect the selective allocation of attentional resources (Isreal et al., 1980). A larger amplitude P3 following a larger amplitude N2 therefore makes sense regarding more effective processing of interference and conflict. In the present study, the HA group showed a significantly smaller P3 in response to the far-congruent (least conflict) condition and this finding was not replicated in the low-active group. This shows that the HA group was able to differentiate between different levels of interference to a greater degree than their low-active counterparts. These findings are in accordance with multiple studies demonstrating a positive association between cardiorespiratory fitness and P3 amplitude during cognitive control and attention tasks (Kao et al., 2020). The P3 was modulated in response to task conditions to a greater degree in active vs. low-active participants, demonstrating some association of HA with P3 amplitude. Collectively, evidence from the N2 and P3 ERP components suggests that the HA group was more able to differentially process the levels of conflict during the task.

### Possible Mechanisms

Aerobic exercise seems to influence processes in the nervous system supporting change due to experience (Hötting and Röder, 2013; Raichlen et al., 2016; Raichlen and Alexander, 2017). Endurance exercise leads to angiogenesis in the human brain, increasing cerebral blood flow and neurotrophic factors such as insulin-like growth factor 1 (IGF-1) and brain derived neurotrophic factor (BDNF; Themanson et al., 2008; Jeon and Ha, 2017). BDNF is especially important for both early and late stages of long-term potentiation (LTP; Gottmann et al., 2009) through various intracellular mechanisms (Tanaka et al., 2008; Bosch et al., 2014; Leal et al., 2014; Panja and Bramham, 2014). Aside from increases in BDNF and IGF-1 (Jeon and Ha, 2017; Thacker et al., 2019), moderate intensity exercise (>50% VO_2_max) can increase levels of dopamine, norepinephrine, and glutamate (Meeusen et al., 1997; Lin and Kuo, 2013) and reduce levels of serotonin (Lin and Kuo, 2013) and cortisol (Jeon and Ha, 2017). These factors collectively work to flexibly facilitate and orient LTP and long-term depression of synapses in order to modulate cortical activity. It is evident that aerobic exercise balances the levels of neuromodulators in such a way that primes the cortical environment favorably for learning and consolidation.

Endurance exercise evokes complex behaviors related to both motor and cognitive functions. Whether outside, at home or in the gym; individuals exercise in complex, changing environments. Navigating and responding to these environments during exercise engages multiple components of executive functioning including inhibitory control, action monitoring and attentional switching, in addition to motor control (Raichlen and Alexander, 2017). Furthermore, during endurance exercise, both external environmental (context, competitors, location, etc.) and internal factors such as motivation, affect, fatigue, physiological signals, and experiential knowledge must be integrated to modify the intensity or pace and ultimately to decide when (or when not) to terminate the exercise (Robertson and Marino, 2016). A role for the prefrontal cortex in this regard has been proposed by Robertson and Marino (2016) in which the lateral PFC processes input from the ACC and orbitofrontal cortex to achieve an appropriate response *via* motor systems such as the premotor area and the basal ganglia. This concept reinforces the notion of an actively engaged PFC during endurance exercise. Given that aerobic exercise creates an ideal environment for use-dependent plasticity and learning, it is reasonable to believe that the cognitive networks active during said exercise will be strengthened through practice. The habitual combination of favorable neuromodulator levels and cognitive engagement therefore presents one way in which chronic aerobic exercise may enhance executive functions including cognitive control and conflict monitoring.

### Limitations

The assignment to the HA or low-active group was based on self-reported results of the IPAQ (2002), results may therefore be biased by memory error. Though EEG provides excellent temporal resolution, the spatial resolution is weak; limiting the ability to investigate with confidence the specific brain regions contributing to any neuroelectric differences. Given the proposed mechanism by which exercise may enhance cognition, the modality of habitual aerobic exercise may become relevant. Therefore, the heterogeneity of the HA group could reflect a limitation. Participants participated in activities such as running, cross-country skiing, hockey, ringette, and triathlon; these different activities could demand different levels of executive function potentially leading to different patterns of use-dependent plasticity. Furthermore, though the vast majority of participants were students at the University of Waterloo, reflecting a generally homogenous sample regarding education and relative socio-economic status, we cannot ignore that factors aside from activity level can and will impact individuals’ cognitive ability and performance on cognitive tasks which we cannot account for. The relatively low sample size has an inherent risk of overestimation of large effect sizes. Finally, when investigating the effects of exercise in a non-longitudinal format it is inherently difficult to distinguish nature from nurture. While the HA group may show modulation of certain ERP components due to chronic aerobic exercise; the possibility exists that pre-existing modulation of ERP components predisposes these individuals to partake in chronic aerobic exercise.

## Conclusion

The present study has contributed to a growing body of literature describing the influence of chronic aerobic exercise on cognition. We found numerous electrophysiological differences between a group of chronically aerobically active individuals and their low-active counterparts. Collectively these findings show that the HA group was more receptive to the level of conflict displayed, as described by electrophysiological indices; high conflict trials resulted in a larger amplitude N2, while lower conflict trials resulted in a lower amplitude P3 and a lower probability of committing an error in the HA, but not the low-active group. These findings provide evidence that HA may confer some endogenous cognitive benefit regarding performance and conflict monitoring and the allocation of cognitive control according to task demands.

## Data Availability Statement

The datasets generated for this study are available on request to the corresponding author.

## Ethics Statement

The studies involving human participants were reviewed and approved by Research Ethics Board, University of Waterloo. The patients/participants provided their written informed consent to participate in this study.

## Author Contributions

MW and WS contributed to the conception and design of the study. MW collected and analyzed the data and wrote the manuscript. All authors contributed to the article and approved the submitted version.

## Funding

This work was supported by a research grant (RGPIN-2019-04414) to WS from the National Sciences and Engineering Research Council of Canada (NSERC).

## Conflict of Interest

The authors declare that the research was conducted in the absence of any commercial or financial relationships that could be construed as a potential conflict of interest.

## Publisher’s Note

All claims expressed in this article are solely those of the authors and do not necessarily represent those of their affiliated organizations, or those of the publisher, the editors and the reviewers. Any product that may be evaluated in this article, or claim that may be made by its manufacturer, is not guaranteed or endorsed by the publisher.
